# Supplementary material for: GSK3β Inhibition Is the Molecular Pivot That Underlies the Mir-210-Induced Attenuation of Intrinsic Apoptosis Cascade during Hypoxia
Source: Int J Mol Sci. 2022 Aug 19;23(16):9375. doi: 10.3390/ijms23169375 (PMC9409400; doi:10.3390/ijms23169375)
Supplement: Supplementary file 1 [file ijms-23-09375-s001.zip › ijms-1819501-supplementary.pdf]

Supplementary Data and Materials

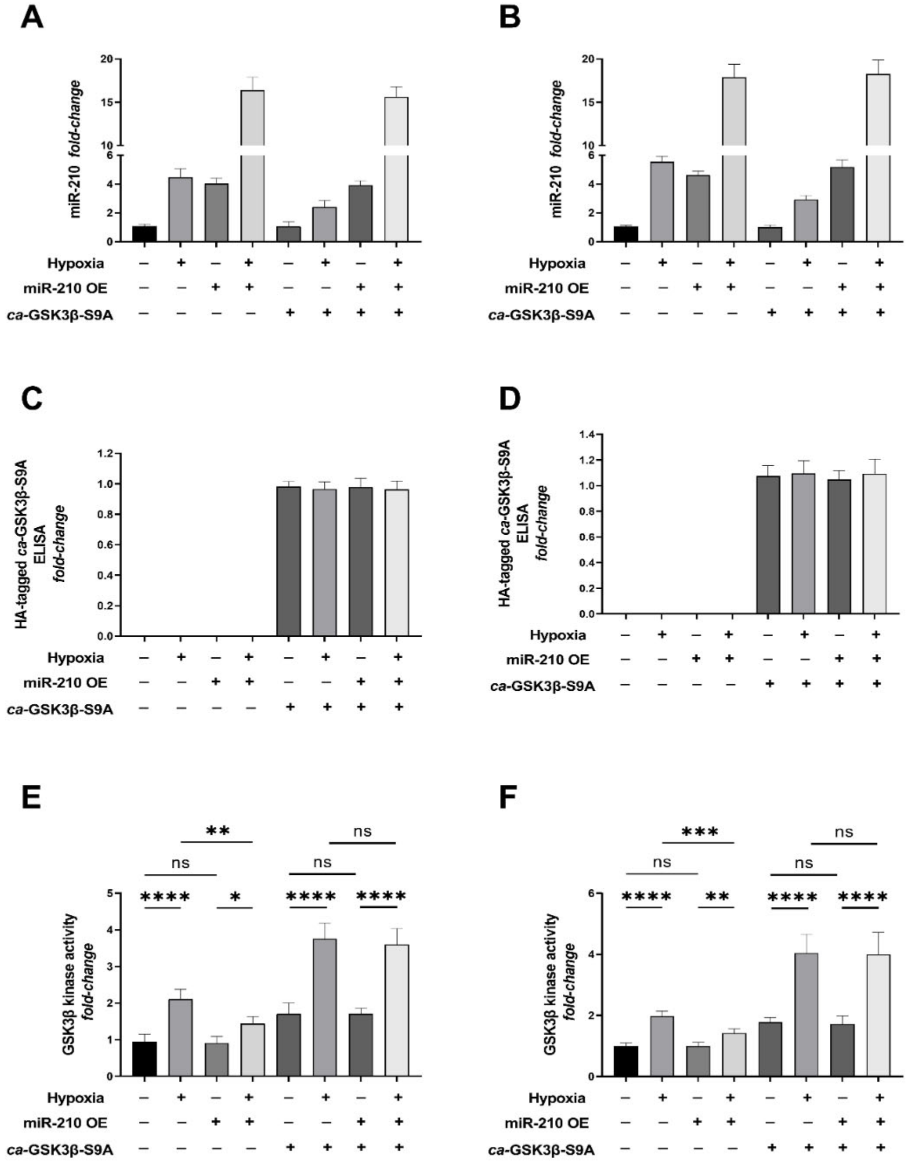

Supplementary Figure S1: **Validation of miR-210 overexpression and ectopic expression of the HA-tagged *ca*-GSK3β-S9A mutant, as well as the determination of GSK3β kinase activity in *native* lysates subjected to quantitative sandwich ELISA immunoassays and caspase-3 activity assay.** (A,B) miR-210 hybridization immunoassay validating miR-210 overexpression in the respective *native* lysates subjected to quantitative sandwich ELISA immunoassays (A) and caspase-3 activity assay (B). (C,D) ELISA immunoassays executed against the HA-tag validating the ectopic expression of the HA-tagged *ca*-GSK3β-S9A mutant in the respective *native* lysates subjected to quantitative sandwich ELISA immunoassays (C) and caspase-3 activity assay (D). (E,F) GSK3β kinase activity assay validating the translative effect of ectopically expressing the HA-tagged *ca*-GSK3β-S9A mutant in the respective *native* lysates subjected to quantitative sandwich ELISA immunoassays (E) and caspase-3 activity assay (F). All data is expressed as *Mean* ± *S.D fold-change* from three (3) technical replicates for each of the four biological replicates belonging to each experimental group (n=4).
*** $p \leq 0.05$; ** $p \leq 0.01$; *** $p \leq 0.001$; **** $p \leq 0.0001$; ns: not significant ($p > 0.05$)**
OE: miR-210 overexpression; S.D: standard deviation.

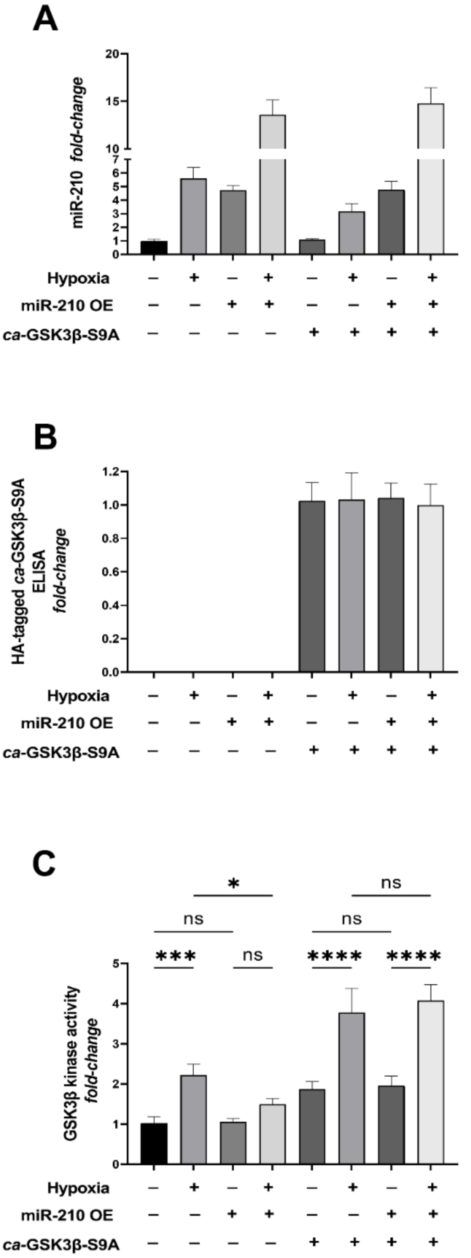

Supplementary Figure S2: **Validation of miR-210 overexpression and ectopic expression of the HA-tagged *ca*-GSK3β-S9A mutant, as well as the determination of GSK3β kinase activity in *native* lysates subjected to DFF40 endonuclease activity assay.** (A) miR-210 hybridization immunoassay validating miR-210 overexpression in the respective *native* lysates subjected to DFF40 endonuclease activity assay. (B) ELISA immunoassay executed against the HA-tag validating the ectopic expression of the HA-tagged *ca*-GSK3β-S9A mutant in the respective *native* lysates subjected to TUNEL assay and DFF40 endonuclease activity assay. (C) GSK3β kinase activity assay validating the translative effect of ectopically expressing the HA-tagged *ca*-GSK3β-S9A mutant in the respective *native* lysates subjected to TUNEL assay and DFF40 endonuclease activity assay. All data is expressed as *Mean ± S.D fold-change* from three (3) technical replicates for each of the four biological replicates belonging to each experimental group
(n=4).

*** $p \leq 0.05$; *** $p \leq 0.001$; **** $p \leq 0.0001$; ns: not significant ($p > 0.05$)**

OE: miR-210 overexpression; S.D: standard deviation.

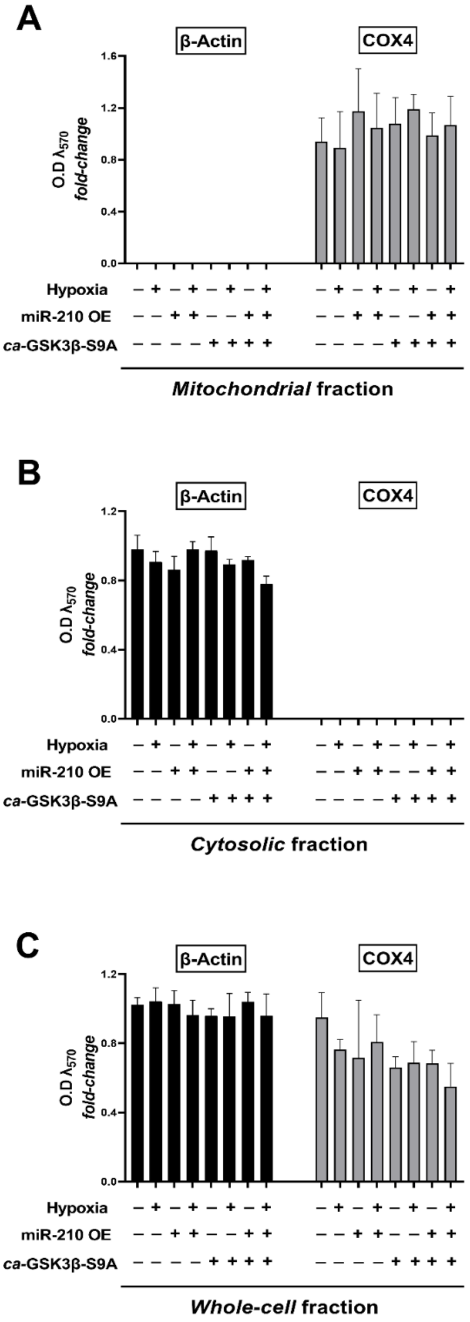

Supplementary Figure S3: Validity and integrity of the fractionated *mitochondrial* and *cytosolic* compartments subjected to *Cytochrome C* release assay and quantitative ELISA immunoassays determining the abundance of the BCL2 family of proapoptotic and antiapoptotic proteins. The integrity of the mitochondrial fraction was validated by the presence of COX4 concomitant with the absence of β-Actin, while the integrity of the cytosolic fraction was validated by the absence of COX4 concomitant with the presence of β-Actin. (A-C) Quantitative sandwich ELISA immunoassays, determining the presence and relative abundance of COX4 and β-Actin in the mitochondrial fraction (A), cytosolic fraction (B), as well as the whole-cell lysates (C), demonstrate the fractional integrity and validity of the respective subcellular compartments. All data is expressed as *Mean ± S.D fold-change* from three (3) technical replicates for each of the four biological replicates belonging to each experimental group (n=4).

OE: miR-210 overexpression; O.D: optical density; S.D: standard deviation.

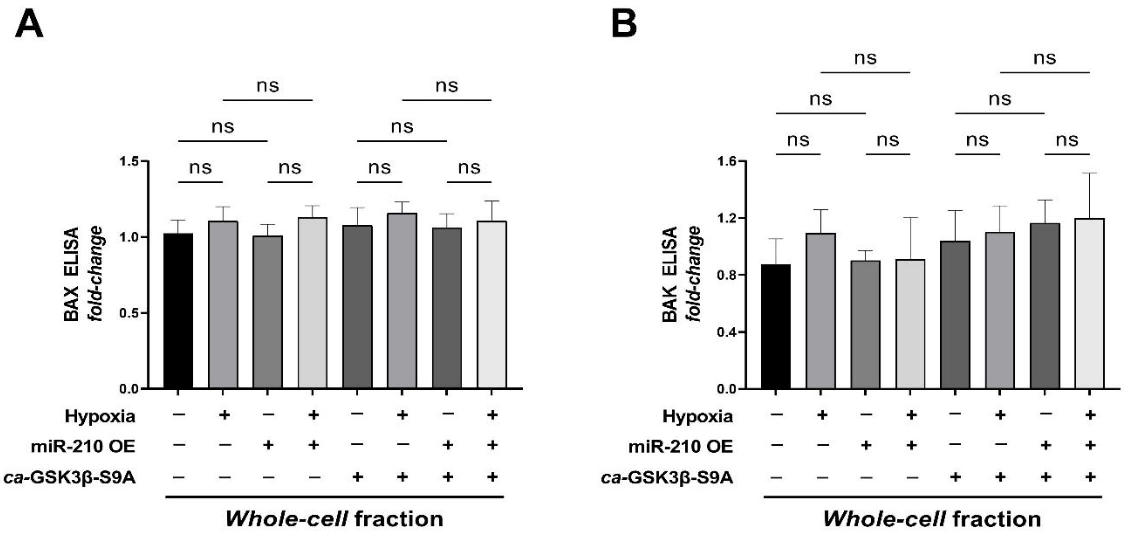

Supplementary Figure S4: (A,B) Quantitative ELISA immunoassays determining the abundance of BAX (A) and BAK (B) in the respective whole-cell lysates. All data is expressed as *Mean ± S.D fold-change* from three (3) technical replicates for each of the four biological replicates belonging to each experimental group (n=4).
ns: not significant (p > 0.05)
OE: miR-210 overexpression; S.D: standard deviation.

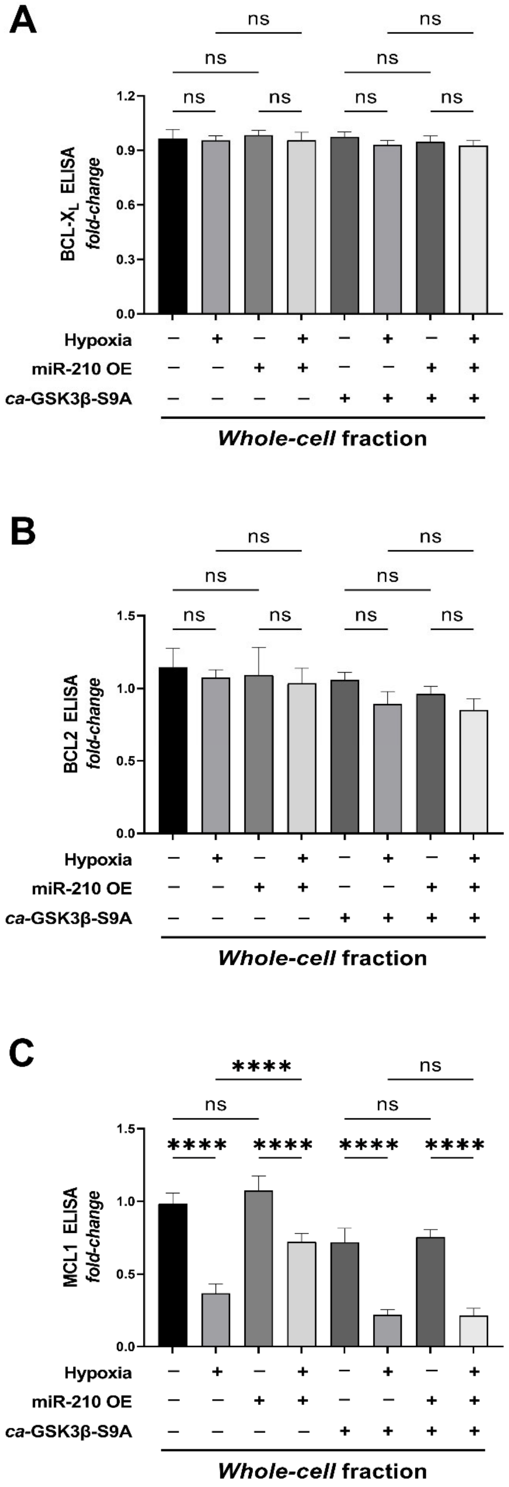

Supplementary Figure S5: (A-C) Quantitative ELISA immunoassays determining the abundance of the BCL2 family of antiapoptotic proteins, BCL-X$_L$ (A), BCL2 (B), and MCL1 (C), in the respective whole-cell lysates. All data is expressed as *Mean* ± *S.D fold-change* from three (3) technical replicates for each of the four biological replicates belonging to each experimental group (n=4).

****** p ≤ 0.0001; ns: not significant (p > 0.05)**

OE: miR-210 overexpression; S.D: standard deviation.

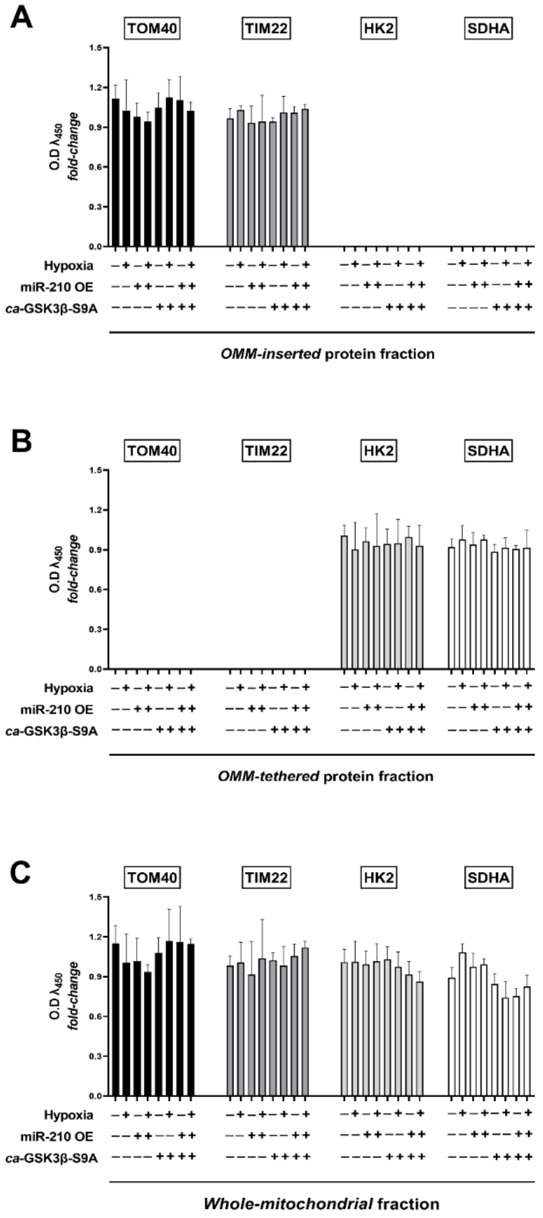

Supplementary Figure S6: Validation of the integrity of the alkali-resistant *OMM-inserted* (OMM-embedded) protein fraction and the alkali-soluble *OMM-tethered* (OMM-anchored) protein fraction. The integrity of the *OMM-inserted* protein fraction was validated by the presence of TOM40 and TIM22 concomitant with the absence of SDHA and HK2. The integrity of the *OMM-tethered* protein fraction was validated by the absence of TOM40 and TIM22 concomitant with the presence of SDHA and HK2. (A-C) Quantitative sandwich ELISA immunoassays, determining the presence and relative abundance of TOM40, TIM22, SDHA, and HK2 in the *OMM-inserted* protein fraction (A), *OMM-tethered* protein fraction (B), as well as the whole-mitochondrial fraction (C), demonstrate the fractional integrity and validity of the respective mitochondrial compartments. OE: miR-210 overexpression; O.D: optical density; S.D: standard deviation; OMM: outer mitochondrial membrane.

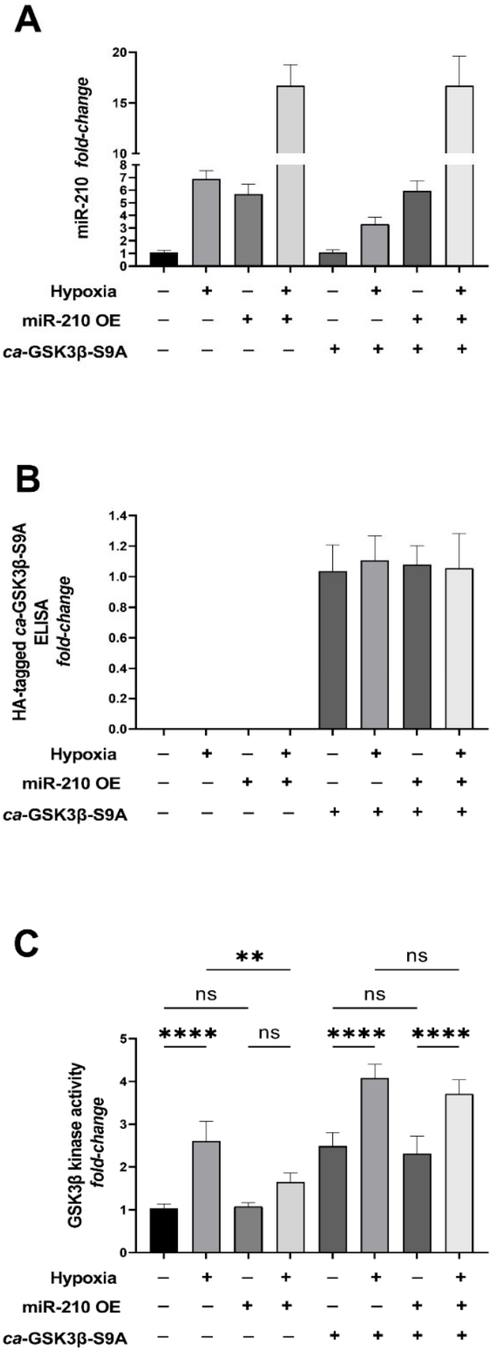

Supplementary Figure S7: **Validation of miR-210 overexpression and ectopic expression of the HA-tagged *ca*-GSK3β-S9A mutant, as well as the determination of GSK3β kinase activity in the cellular *inputs* that engendered the *OMM-inserted* protein fraction and the *OMM-tethered* protein fraction.** (A) miR-210 hybridization immunoassay validating miR-210 overexpression in the respective in the cellular *inputs* that engendered the *OMM-inserted* protein fraction and the *OMM-tethered* protein fraction. (B) ELISA immunoassay executed against the HA-tag validating the ectopic expression of the HA-tagged *ca*-GSK3β-S9A mutant in the respective in the cellular *inputs* that engendered the *OMM-inserted* protein fraction and the *OMM-tethered* protein fraction. (C) GSK3β kinase activity assay validating the translative effect of ectopically expressing the HA-tagged *ca*-GSK3β-S9A mutant in the respective in the cellular *inputs* that engendered the *OMM-inserted* protein fraction and the *OMM-tethered* protein fraction. All data is expressed as *Mean ± S.D fold-change* from three (3) technical replicates for each of the four biological replicates belonging to each experimental group (n=4).

**** $p \leq 0.01$; **** $p \leq 0.0001$; ns: not significant ($p > 0.05$)**

OE: miR-210 overexpression; S.D: standard deviation.

Supplementary Table S1: Composition of the *hypoxia medium*

**Component**	**500 mL**	**Final concentration**	**Source (Notation)**
DMEM, No Glucose	464.45 mL	93% v/v	1
Creatine	131.2 mg	5 mM	2
D-(+)-Glucose Solution 2.5 M, 450 g/L	0.55 mL	2.75 mM	3
Glutamine 200 mM	5 mL	2 mM	4
HEPES 1M	5 mL	10 mM	5
L-Carnitine, 200 mM	5 mL	2 mM	6
Non-essential Amino Acids, 100x	5 mL	N/A*	7
Sodium Pyruvate 100 mM	5 mL	1 mM	8
Taurine 500 mM	5 mL	5 mM	9
Linoleic Acid-Oleic Acid-Albumin, 100x	5 mL	N/A*	10

Supplementary Table S1: Notation legend

[1] Thermo Fisher Scientific, Oslo, Norway, Catalogue # 11966025

[2] Sigma Aldrich / Merck Millipore / Merck Life Science, Darmstadt, Germany, Catalogue # C3630-100G

[3] Sigma Aldrich / Merck Millipore / Merck Life Science, Oslo, Norway, Catalogue # G8769

[4] Thermo Fisher Scientific, Oslo, Norway, Catalogue # A2916801

[5] Sigma Aldrich / Merck Millipore / Merck Life Science, Darmstadt, Germany, Catalogue # H4034-500G

[6] Sigma Aldrich / Merck Millipore / Merck Life Science, Darmstadt, Germany, Catalogue # C0283-25G

[7] Thermo Fisher Scientific, Oslo, Norway, Catalogue # 11140035

[8] Thermo Fisher Scientific, Oslo, Norway, Catalogue # 11360070

[9] Sigma Aldrich / Merck Millipore / Merck Life Science, Darmstadt, Germany, Catalogue # T8691-100G

[10] Sigma Aldrich / Merck Millipore / Merck Life Science, Darmstadt, Germany, Catalogue # L9655-5ML

* N/A - Not Applicable
